# Connecting the data landscape of long‐term ecological studies: The SPI‐Birds data hub

**DOI:** 10.1111/1365-2656.13388

**Published:** 2020-12-04

**Authors:** Antica Culina, Frank Adriaensen, Liam D. Bailey, Malcolm D. Burgess, Anne Charmantier, Ella F. Cole, Tapio Eeva, Erik Matthysen, Chloé R. Nater, Ben C. Sheldon, Bernt‐Erik Sæther, Stefan J. G. Vriend, Zuzana Zajkova, Peter Adamík, Lucy M. Aplin, Elena Angulo, Alexandr Artemyev, Emilio Barba, Sanja Barišić, Eduardo Belda, Cemal Can Bilgin, Josefa Bleu, Christiaan Both, Sandra Bouwhuis, Claire J. Branston, Juli Broggi, Terry Burke, Andrey Bushuev, Carlos Camacho, Daniela Campobello, David Canal, Alejandro Cantarero, Samuel P. Caro, Maxime Cauchoix, Alexis Chaine, Mariusz Cichoń, Davor Ćiković, Camillo A. Cusimano, Caroline Deimel, André A. Dhondt, Niels J. Dingemanse, Blandine Doligez, Davide M. Dominoni, Claire Doutrelant, Szymon M. Drobniak, Anna Dubiec, Marcel Eens, Kjell Einar Erikstad, Silvia Espín, Damien R. Farine, Jordi Figuerola, Pınar Kavak Gülbeyaz, Arnaud Grégoire, Ian R. Hartley, Michaela Hau, Gergely Hegyi, Sabine Hille, Camilla A. Hinde, Benedikt Holtmann, Tatyana Ilyina, Caroline Isaksson, Arne Iserbyt, Elena Ivankina, Wojciech Kania, Bart Kempenaers, Anvar Kerimov, Jan Komdeur, Peter Korsten, Miroslav Král, Miloš Krist, Marcel Lambrechts, Carlos E. Lara, Agu Leivits, András Liker, Jaanis Lodjak, Marko Mägi, Mark C. Mainwaring, Raivo Mänd, Bruno Massa, Sylvie Massemin, Jesús Martínez‐Padilla, Tomasz D. Mazgajski, Adèle Mennerat, Juan Moreno, Alexia Mouchet, Shinichi Nakagawa, Jan‐Åke Nilsson, Johan F. Nilsson, Ana Cláudia Norte, Kees van Oers, Markku Orell, Jaime Potti, John L. Quinn, Denis Réale, Tone Kristin Reiertsen, Balázs Rosivall, Andrew F Russell, Seppo Rytkönen, Pablo Sánchez‐Virosta, Eduardo S. A. Santos, Julia Schroeder, Juan Carlos Senar, Gábor Seress, Tore Slagsvold, Marta Szulkin, Céline Teplitsky, Vallo Tilgar, Andrey Tolstoguzov, János Török, Mihai Valcu, Emma Vatka, Simon Verhulst, Hannah Watson, Teru Yuta, José M. Zamora‐Marín, Marcel E. Visser

**Affiliations:** ^1^ Department of Animal Ecology Netherlands Institute of Ecology (NIOO‐KNAW) Wageningen The Netherlands; ^2^ Department of Biology University of Antwerp Antwerp Belgium; ^3^ Department of Evolutionary Genetics Leibniz Institute for Zoo and Wildlife Research Berlin Germany; ^4^ RSPB Centre for Conservation Science The Lodge Sandy UK; ^5^ Centre for Research in Animal Behaviour University of Exeter Exeter UK; ^6^ CEFE University of Montpellier CNRS EPHE IRD University of Paul Valéry Montpellier 3 Montpellier France; ^7^ Edward Grey Institute Department of Zoology University of Oxford Oxford UK; ^8^ Department of Biology University of Turku Turku Finland; ^9^ Kevo Subarctic Research Institute University of Turku Turku Finland; ^10^ Centre for Biodiversity Dynamics Department of Biology Norwegian University of Science and Technology Trondheim Norway; ^11^ Department of Zoology Faculty of Science Palacký University Olomouc Czech Republic; ^12^ Cognitive and Cultural Ecology Research Group Max Planck Institute of Animal Behavior Radolfzell Germany; ^13^ Centre for the Advanced Study of Collective Behaviour University of Konstanz Konstanz Germany; ^14^ Department of Ethology and Biodiversity Conservation Estación Biológica de Doñana‐CSIC Seville Spain; ^15^ Institute of Biology of the Karelian Research Centre of the Russian Academy of Sciences Petrozavodsk Russia; ^16^ Cavanilles Institute of Biodiversity and Evolutionary Biology University of Valencia Paterna Spain; ^17^ Institute of Ornithology Croatian Academy of Sciences and Arts Zagreb Croatia; ^18^ Universitat Politècnica de València Valencia Spain; ^19^ Biodiversity and Conservation Lab Department of Biology METU Ankara Turkey; ^20^ Université de Strasbourg CNRS IPHC UMR 7178 Strasbourg France; ^21^ Groningen Institute for Evolutionary Life Sciences University of Groningen Groningen; ^22^ Institute of Avian Research Wilhelmshaven Germany; ^23^ Institute of Biodiversity, Animal Health & Comparative Medicine University of Glasgow Glasgow UK; ^24^ Department of Animal & Plant Sciences University of Sheffield Sheffield UK; ^25^ Department of Vertebrate Zoology Faculty of Biology Lomonosov Moscow State Univ Moscow Russia; ^26^ Department of Biology Lund University Lund Sweden; ^27^ Stazione Ornitologica Monreale Italy; ^28^ Department STEBICEF Università degli Studi di Palermo Palermo Italy; ^29^ Centre for Ecological Research Institute of Ecology and Botany Vácrátót Hungary; ^30^ Station d’Ecologie Théorique et Expérimentale du CNRS (UMR5321) Moulis France; ^31^ Institute of Environmental Sciences Jagiellonian University Kraków; ^32^ Evolutionary Physiology Group Max Planck Institute for Ornithology Seewiesen Germany; ^33^ Laboratory of Ornithology Cornell University Ithaca NY USA; ^34^ Behavioural Ecology Department of Biology Ludwig‐Maximilians University of Munich Planegg‐Martinsried Germany; ^35^ CNRS Department of Biometry & Evolutionary Biology University Lyon 1, University of Lyon Villeurbanne France; ^36^ Department of Ecology and Genetics and Animal Ecology Uppsala University Uppsala Sweden; ^37^ Institute of Environmental Sciences Jagiellonian University Krakow Poland; ^38^ Evolution & Ecology Research Centre and School of Biological, Earth and Environmental Sciences University of New South Wales Sydney Australia; ^39^ Museum and Institute of Zoology Polish Academy of Sciences Warsaw Poland; ^40^ Norwegian Institute for Nature Research FRAM – High North Research Centre for Climate and the Environment Tromsø Norway; ^41^ Area of Toxicology Department of Health Sciences University of Murcia Murcia Spain; ^42^ Department of Collective Behavior Max Planck Institute of Animal Behavior Konstanz Germany; ^43^ Department of Biology University of Konstanz Konstanz Germany; ^44^ Department of Wetland Ecology Estación Biológica de Doñana (CSIC) Sevilla Spain; ^45^ Environmental Engineering Department Hacettepe University Ankara Turkey; ^46^ Lancaster Environment Centre Lancaster University Lancaster UK; ^47^ Behavioural Ecology Group Department of Systematic Zoology and Ecology ELTE Eötvös Loránd University Budapest Hungary; ^48^ Department of integrative Biology and Biodiversity Research University of Natural Resources and Life Sciences Vienna Vienna Austria; ^49^ Behavioural Ecology Group Biological Sciences Anglia Ruskin University Cambridge UK; ^50^ Zvenigorod Biological Station Faculty of Biology Lomonosov Moscow State University Moscow Russia; ^51^ Ornithological Station, Museum and Institute of Zoology Polish Academy of Sciences Gdańsk Poland; ^52^ Department Behavioural Ecology & Evolutionary Genetics Max Planck Institute for Ornithology Seewiesen Germany; ^53^ Department of Animal Behaviour Bielefeld University Bielefeld Germany; ^54^ Department of Zoology University of Otago Dunedin New Zealand; ^55^ Department of Nature Conservation Environmental Board Estonia; ^56^ MTA‐PE Evolutionary Ecology Research Group University of Pannonia Veszprém Hungary; ^57^ Department of Zoology Institute of Ecology and Earth Sciences University of Tartu Tartu Estonia; ^58^ Division of Biological Sciences University of Montana Missoula USA; ^59^ Department of Biodiversity Conservation and Ecosystem Restoration Pyrenean Institute of Ecology (CSIC) Jaca Spain; ^60^ Department of Biological Sciences University of Bergen Bergen Norway; ^61^ Department de Ecología Evolutiva Museo Nacional de Ciencias Naturales (CSIC) Madrid Spain; ^62^ Department of Life Sciences MARE ‐ Marine and Environmental Sciences Centre University of Coimbra Coimbra Portugal; ^63^ Behavioural Ecology Group Department of Animal Sciences Wageningen University & Research Wageningen The Netherlands; ^64^ Ecology and Genetics Research Unit University of Oulu Oulu Finland; ^65^ School of Biological, Earth and Environmental Sciences University College Cork Cork Ireland; ^66^ Département des Sciences Biologiques Université du Québec A Montréal Montréal Canada; ^67^ Centre for Ecology and Conservation University of Exeter Penryn Cornwall UK; ^68^ BECO do Departamento de Zoologia Universidade de São Paulo São Paulo Brazil; ^69^ Department of Life Sciences Imperial College London Ascot UK; ^70^ Evolutionary and Behavioural Ecology Research Unit Museu de Ciències Naturals de Barcelona Barcelona Spain; ^71^ Centre for Ecological and Evolutionary Synthesis (CEES) Department of Biosciences University of Oslo Oslo Norway; ^72^ Centre of New Technologies University of Warsaw Warsaw Poland; ^73^ Ecological Genetics Research Unit, Organismal and Evolutionary Biology Research Programme Faculty of Biological & Environmental Sciences University of Helsinki Helsinki Finland; ^74^ Yamashina Institute for Ornithology Abiko Japan; ^75^ Graduate School of Environment Science Hokkaido University Sapporo Japan; ^76^ Department of Zoology and Physical Anthropology University of Murcia Murcia Spain

**Keywords:** birds, data standards, database, FAIR data, long‐term studies, meta‐data standards, research network

## Abstract

1. The integration and synthesis of the data in different areas of science is drastically slowed and hindered by a lack of standards and networking programmes. Long‐term studies of individually marked animals are not an exception. These studies are especially important as instrumental for understanding evolutionary and ecological processes in the wild. Furthermore, their number and global distribution provides a unique opportunity to assess the generality of patterns and to address broad‐scale global issues (e.g. climate change).
2. To solve data integration issues and enable a new scale of ecological and evolutionary research based on long‐term studies of birds, we have created the SPI‐Birds Network and Database (www.spibirds.org)—a large‐scale initiative that connects data from, and researchers working on, studies of wild populations of individually recognizable (usually ringed) birds. Within year and a half since the establishment, SPI‐Birds has recruited over 120 members, and currently hosts data on almost 1.5 million individual birds collected in 80 populations over 2,000 cumulative years, and counting.
3. SPI‐Birds acts as a data hub and a catalogue of studied populations. It prevents data loss, secures easy data finding, use and integration and thus facilitates collaboration and synthesis. We provide community‐derived data and meta‐data standards and improve data integrity guided by the principles of Findable, Accessible, Interoperable and Reusable (FAIR), and aligned with the existing metadata languages (e.g. ecological meta‐data language).
4. The encouraging community involvement stems from SPI‐Bird's decentralized approach: research groups retain full control over data use and their way of data management, while SPI‐Birds creates tailored pipelines to convert each unique data format into a standard format. We outline the lessons learned, so that other communities (e.g. those working on other taxa) can adapt our successful model. Creating community‐specific hubs (such as ours, COMADRE for animal demography, etc.) will aid much‐needed large‐scale ecological data integration.

## Graphical abstract

SPI‐Birds is a global‐scale initiative that creates meta‐data and data standards for data collected in populations of uniquely marked individuals that are followed over years. The authors host data on nearly 1.5 million birds collected over cumulative 2000 years. Everyone can search for populations and request their data via SPI‐Birds database.

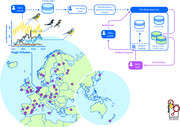

## 1 INTRODUCTION

### 1.1 The importance of long‐term individual‐based studies

Long‐term individual‐based studies of animals in their natural environment underpin our understanding of evolutionary and ecological patterns and processes in wild populations (Clutton‐Brock & Sheldon, 2010). These studies considerably increase our ability to establish the links among genes, individual traits (including physiology and behaviour), fitness and the environment (Bonnet et al., 2019; Broggi et al., 2005; Johnston et al., 2016; Schroeder et al., 2015). They further document the responses of natural populations to changing environments (Espín et al., 2016; Grant & Grant, 2002; Mennerat et al., 2019; Paniw et al., 2019; Réale et al., 2003), and facilitate evidence‐based conservation (Festa‐Bianchet et al., 2019; Tylianakis et al., 2008; Vatka et al., 2014).

The first large‐scale individual‐based field studies of vertebrates were conducted on birds and birds remain the most commonly studied group (Clutton‐Brock & Sheldon, 2010; Radchuk et al., 2019). Several types of birds (e.g. hole‐nesting passerines, colonially breeding seabirds or fairy‐wrens) have proven to be highly suitable for long‐term individual‐based monitoring of reproduction and survival. Some of the longest‐running field studies with over 65 years of non‐interrupted time series focus on hole‐nesting birds (e.g. Ahola et al., 2007; Kluijver, 1951; Lack, 1954, 1966). Hole‐nesters are well suited to detailed study as they often breed at high densities in nest‐boxes (Dhondt, 2007; Lambrechts et al., 2010), which allows for easy monitoring of the breeding performance (e.g. lay date, clutch size, nesting success) and capture of a large number of birds. Up to now studies cover species with different life histories over a wide latitudinal and longitudinal range, and in a variety of habitat types, including urban habitats (Andersson et al., 2015; Charmantier et al., 2017; Corsini et al., 2017; Senar et al., 2017; Seress et al., 2018). Importantly, these long‐term datasets make it possible to answer questions that were not anticipated at the onset of data collection (e.g. influence of global warming on phenology, Visser et al., 1998; effects of habitat fragmentation, Dhondt, 2007).

The main asset of individual‐based bird studies is not only the long temporal scale, but also the high degree of spatial replication provided by multiple studies conducted simultaneously (Dingemanse et al., 2012; Korsten et al., 2010). The amount of information available when studies are combined has the potential to bring our understanding of ecological and evolutionary processes to entirely new levels, and has, not surprisingly, led to a number of collaborative projects (e.g. Both et al., 2004; Eeva et al., 2011; Keogan et al., 2018; Laine et al., 2016; Loukola et al., 2020; Sæther et al., 2007; Samplonius et al., 2018; Vaugoyeau et al., 2016; Wilkins et al., 2016), and we provide some examples in more detail in Box 1. This large‐scale synthesis (including the meta‐analysis context, Culina, Crowther, et al., 2018; Siepielski et al., 2017, 2019) is especially important for capturing the diversity of biological systems and the variation in ecological conditions that are experienced by different populations. Which processes may be described as being general? Which processes can be identified as being more specific to certain environmental conditions? Only when we have answers to these questions, we can make predictions and tackle global issues, such as habitat degradation, animal welfare or global warming, and gain insights into reproducibility of findings based on ecological time series.

BOX 1 **Examples of using multiple wild populations**

**(a) Assessing the ability to substitute space‐for‐time**

Within the scope of understanding and predicting ecological and evolutionary responses to climate change, sampling and studying multiple populations of the same species across latitudinal or altitudinal gradient may provide insights into adaptation to climate variation, if we assume that time can be substituted by space in the processes involved (Blois et al., 2013; Phillimore et al., 2010). For example, Bay et al. (2018) sampled yellow warblers *Setophaga petechia* across their breeding range to analyse genomic variation across space and environments (climate, vegetation type and elevation). Assuming that the current spatial variation in traits of this species may provide information on temporal variation in the future, this study suggested that those yellow warbler populations that have already experienced the largest population declines, require the greatest shifts in allele frequencies to keep pace with future climate change (i.e. are most genetically vulnerable). Similarly, urban‐driven evolutionary adaptation is a fascinating process that not only can be followed in time, but also across space, and fostering long‐term ecological and evolutionary monitoring in urban areas is key (Szulkin et al., 2020a). In urban evolutionary biology, the spatial dimension is particularly valuable from an empirical perspective as it allows researchers to take advantage of replicated urbanization gradients, where each city or urban area acts as independent urbanization replicate (Santangelo et al., 2020; Szulkin et al., 2020b; Vaugoyeau et al., 2016).

**(b) Using spatial replication to infer causal relationships**

Spatial variation in local temperature trends across long‐term population studies allows researchers to separate effects of climate change from confounding correlates which may also be changing over time. As we, unfortunately, have no replicate world without climate change, it is often difficult to attribute changes in local phenotypic distributions to temperature change, rather than to the multitude of other environmental changes that may happen simultaneously. For example, based on local trends of spring temperatures and laying dates in 25 long‐term populations of Ficedula flycatchers across Europe, Both et al. (2004) showed that many populations did not exhibit a trend towards earlier breeding, but altogether, there was a clear negative population‐level correlation between the trend in laying date and the trend in temperature. In a similar analysis on great tits *Parus major* and blue tits *Cyanistes caeruleus*, such an effect of local temperature was not found, rather, populations originally having a low frequency of second broods did advance, whereas populations in which second broods used to be common did not advance their laying dates (Visser et al., 2003). These examples nicely illustrate how both within‐ and between‐species comparisons of long‐term studies deepen our understanding of how organisms may adapt to climate change.

**(c) Comparisons of evolutionary potential**

Evolutionary potential depends on the genetic architecture of traits. From a quantitative genetics perspective, this architecture is summarized in G, the additive genetic (co)variance matrix. Comparisons of evolutionary potential across populations or species enable us to evaluate the generality of evolutionary constraints (Agrawal & Stinchcombe, 2009) and to gain insight into the evolution of the underlying genetic architecture (McGlothlin et al., 2018; Steppan et al., 2002). For example, using long‐term datasets with pedigree information, Teplitsky et al. (2014) assessed the expected constraints on evolutionary responses of morphological traits in ten populations of seven wild bird species. Based on estimated G matrices and selection gradients for four morphological traits, their results suggest that genetic correlations may reduce the expected rate of evolution by 28% on average, even for traits such as morphological traits, that are generally thought to have a high evolutionary potential.

In terms of the evolution of genetic architecture, Delahaie et al. (2017) showed that the genetic architecture of life history and morphological traits is relatively conserved across populations of blue tits inhabiting contrasting habitats. Additionally, Martínez‐Padilla et al. (2017) compiled all published estimates of additive genetic variation of morphological traits quantified from 20 long‐term and individually monitored populations of 12 wild European bird species. They found that the evolutionary potential of morphological traits decreases as environmental conditions approaches the extremes, either being favourable or unfavourable. Stronger selection pressures that erode additive genetic variation when environmental conditions were unfavourable or high intraspecific competition in favourable environmental conditions may explain the pattern. These examples illustrate the need of larger scale studies, both in terms of geography and phylogeny, to fully address the question of the evolution of genetic architecture in wild populations.

**(d) Resolving methodological issues**

Long‐term individual level studies often vary in protocols, applied methodologies and approaches to data collection. Using many long‐term datasets may help identify such variation, and point towards those variables that can have potentially significant impacts on how results are interpreted, especially at the between‐study level. Møller et al. (2014) targeted one important, strongly varying component of long‐term hole‐nesting bird studies: nest‐box design. Their study included reproductive data of four bird species: blue and great tits, and pied and collared flycatchers. They have found a positive relationship between nest‐box floor area and clutch size in great tits, and between box material (wood vs. concrete) and clutch size in blue tits. These results indicate that variation in study design at the between‐population level should always be included as it may prove an important predictor of some of the observed inter‐population variation.

Over time, individual‐based studies have become more complex and in addition to data on breeding parameters, other types of data have been collected (e.g. morphological, behavioural, physiological, genetic and genomic). Furthermeor, the number of potential relational links to other sources, such as biological samples, climatic data and individual movement data has increased. With the increasing extent and complexity of datasets we urgently need to address data archiving, standards and integration, not only for individual based‐studies but in all branches where many independent research groups collect similar but differently managed, and consequently under‐exploited, data (the long‐tail of science, Box 2, Wallis et al., 2013). In these branches, transition to Findable, Accessible, Interoperable and Reusable (FAIR) data (Wilkinson, 2016; Box 2) is more urgent, but also more challenging compared to fields where data standards have been set up at the very start (e.g. genomics).

BOX 2 **Glossary**

**Individual‐based studies of birds** – Individual birds are marked with rings engraved with a unique identifying number. Birds are captured (or observed), often over subsequent years, and data on individual characteristics and/or breeding parameters (e.g. laying date, clutch size, number of hatchlings and fledglings, partner) are collected. This information directly links to fitness because it provides data on breeding success and on survival of individuals between years, and thus can be used to study different ecological and evolutionary processes, such as selection on individual traits or population‐dependent processes (e.g. density‐dependent selection). Other types of data are also increasingly collected, for example, behavioural, hormonal, genetic or genomic, fine‐scale environmental data (including e.g. pollutant data).

**Long tail of science** – Dispersed scientific research that is conducted by many individual researchers/teams. Data produced in the long tail tend to be small in volume, and less standardized within the same field of study. The majority of scientific funding is spent on this type of research.

**Open data** – Data that anyone is free to use, reuse and redistribute — subject, at most, to the requirement to attribute or share‐alike, https://creativecommons.org/licenses/by‐sa/2.5/

**FAIR data** – FAIR data are equivalent to open data. FAIR data are structured and described in a way that supports their Findability, Accessibility, Interoperability and Reusability, for both machines and humans.

**Meta‐data** – Data that describe datasets. Meta‐data comprise information explaining the purpose and origin of data, methods used to acquire them, the structure of the data, time references, geographical location, brief description of the study site(s), creator, access conditions and terms of use.

**Data owner** – A person or institution that has collected the raw data and/or is hosting the primary data.

**Data user** – A person interested in using the data owned by the data owner. Data owners can be data users of someone else's data.

**Raw data** – Data as collected in the field.

**Primary data** – Data stored locally by each research group. Primary data might differ from raw data because of (a) errors made during transcribing raw data into primary data or (b) correction of obvious errors in raw data during transcribing them into primary data (c) primary data contain some derivate of primary data (e.g. average value for a repeated measurement of an individual).

**Primary data format** – A format in which primary data are stored. This includes the way that data are divided among different tables, the variables recorded, names of these variables and how values of these variables are expressed.

**Standard data format** – A format agreed upon within the research community to record and archive data. The standard format defines the way data are organized among different tables, the vocabularies used to describe the data elements (names of the variables) and conventions used to express the values of the variables.

**Standard quality check** – A range of checks to test the quality and integrity of the primary data converted into the standard format. Each check differentiates between two main types of flags: ‘warnings’ (values that are uncommon or unusual) and ‘likely errors’ (values that seem impossible).

**Data hub** – A central location to physically store (archive) all data for a certain domain.

**Pipeline** – A set of code functions and commands used to convert data provided in the primary format into the standard format. A pipeline usually has a hierarchical structure (outputs of one component of the processing sequence are fed to the next step) and often is modular (non‐necessary components can be removed or changed to modify the final structure of output data).

Scientific collaborations that involve large‐scale sharing of standardized data, even when access to data is not fully open but restricted, have been shown to generate significant insights, but we can only guarantee this with adequate mechanisms in place to align, store and advertise the data that are available for such endeavours. Examples of projects that successfully integrate animal data across a large number of studies are EURING Data Bank (https://euring.org/, du Feu et al., 2016) that stores encounter records of ringed birds, Movebank database (https://www.movebank.org/, Kranstauber et al., 2011) on animal movement data, and COMADRE database on animal demography (Salguero‐Gómez et al., 2016).

### 1.2 Barriers to collaboration

Ideally, data should be openly archived in a way that supports FAIR principles (Wilkinson, 2016), and as increasingly mandated by funders (Culina, Baglioni, et al., 2018; Roche et al., 2014). All data should be in a single, standard format, and accompanied by rich meta‐data that include the description of the data collection protocols, and support data finding and reuse. In practice, this is difficult to achieve.

The core *cultural/sociological reasons* that prevent open data are the lack of incentives, the fear of being scooped, and worries about losing control and overview over the way the data are interpreted and used (Evans, 2016; Roche et al., 2014). The latter is not without good reason; it is easy to misinterpret data collected under specific ecological conditions and to misunderstand how variables were derived (Mills et al., 2015; Nelson, 2009). Furthermore, even when researchers are willing to adopt common data standards, they might lack the technical knowledge or time. Yet, inspiring examples of overcoming these barriers exist. For example, all national bird ringing schemes originally used their own data storing format, but ultimately agreed on one common output format, creating the European Union for Bird Ringing (EURING, du Feu et al., 2016). Now, all bird ringing data can be brought together at the European level.

The core *practical obstacles* to effective data reuse and collaboration are the lack of: (a) meta‐data standards to describe populations, (b) data standards and (c) a central registry of all the populations (Culina, Baglioni, et al., 2018). To find datasets, researchers commonly search the published literature, then contact data owners (who are not always readily reachable, e.g. if they change institution or retire) to determine whether the data are suited for an intended project and whether their owner is willing to share them. This process can take up to a year, and sometimes it fails (personal experience of the authors). If the data are obtained, the user needs to understand the specific conditions of data collection (e.g. specific field protocols, ecologically relevant conditions), the data structure and vocabularies. Groups/researchers store data in different types of databases and formats, use different vocabularies to name data elements (e.g. different languages) or different coding for the same data element (e.g. some record hatching date as day 1, others as day 0). Thus, data owners usually require much time to extract and compile the data and provide meta‐data to meet the user's needs. This process needs to be repeated for each new collaborative project. Reformatting data is not only time consuming, but may also increase the risk of introducing errors.

Cultural and technical barriers must be solved in parallel, and until open data practices become the norm and researchers recognize their benefits, it is crucial to encourage and enable proper data archiving and establish meta‐data and data standards. To achieve FAIR data, and to increase and facilitate collaboration and data synthesis, we created Studies of Populations of Individuals —Birds (SPI – Birds) Network and Database. To overcome cultural barriers, we opted for an approach where data owners can decide to keep full control over the use of their data or can make their data open access. This approach where some data are open access and some are not has also been previously successful with the Movebank (Kranstauber et al., 2011). Data owners also keep their way of data management (i.e. how they organize their data) and SPI‐Birds converts these primary data into a standard (FAIR) format. All meta‐data stored by SPI Birds are open access, as well as the code to convert primary data into the standard format.

### 1.3 SPI‐birds: Connecting researchers and data

SPI‐Birds Network and Database (www.spibirds.org) is a grassroots initiative that connects researchers working on populations of birds in which individuals are uniquely marked, and thus can be recognized (at capture, or by sight). The main goals are to: (a) increase the coordination and collaboration between research groups; (b) host the registry of populations and equalize the visibility among research groups; (c) buffer against data loss and provide long‐term access to datasets; (d) ensure data quality and integrity; and (e) facilitate data use, and give appropriate credit for data use. To achieve these goals we: (a) derive meta‐data attributes that describe populations (Box 2); (b) centrally archive version‐controlled primary data from research groups, with attached conditions of data use; (c) derive data standards with controlled vocabularies and convert primary data format into a standard format; (d) conduct data quality checks; (e) run a series of technical reports on the impact that protocols for data collection may have on derived variables; (f) provide expert advice to researchers setting up new populations; and (g) provide an online interface to find and request data, and maintain outreach activities.

**Figure 1:**
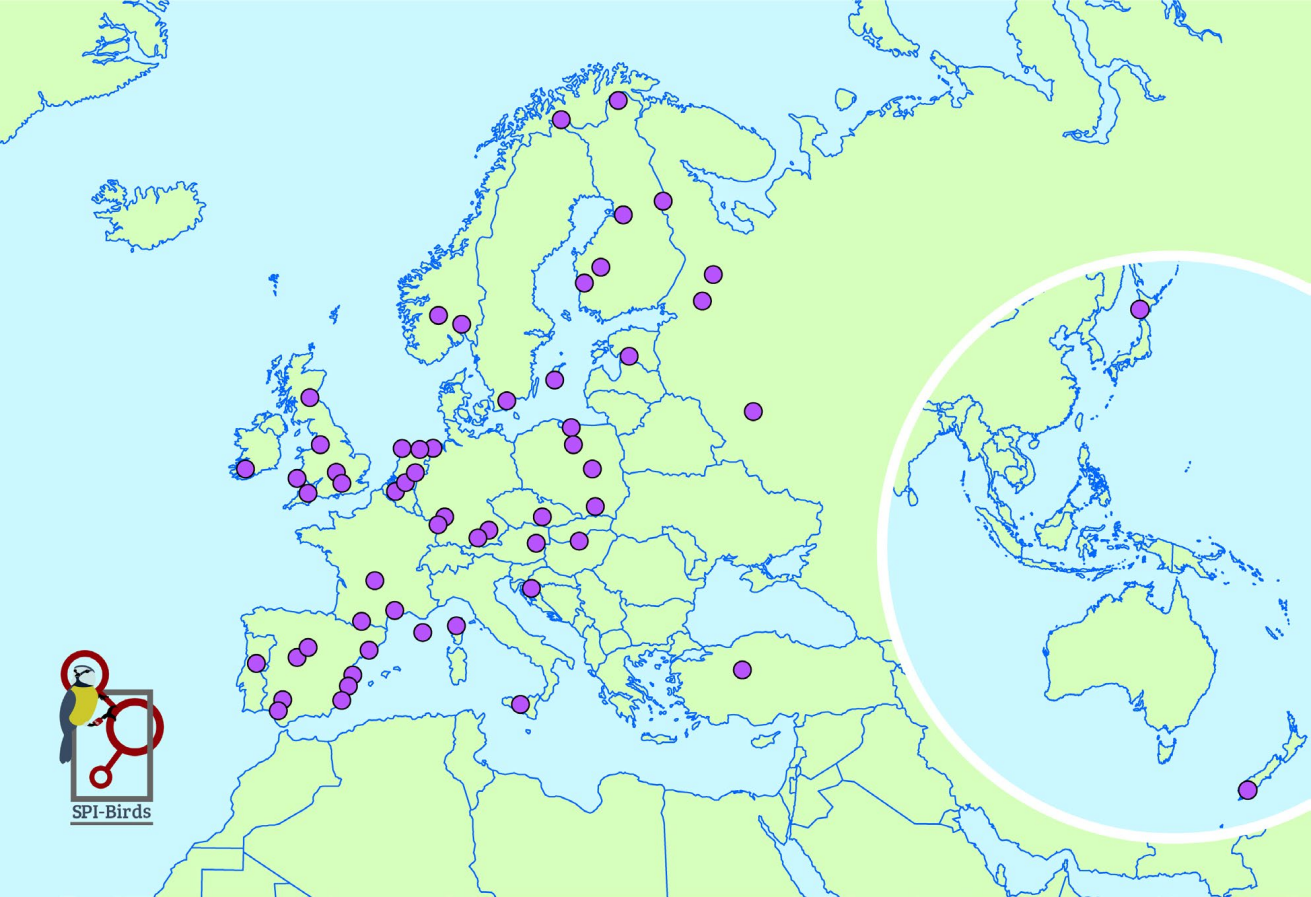
A map showing the location of the populations with the data hosted in the SPI‐Birds database as of August 2020

**Figure 2:**
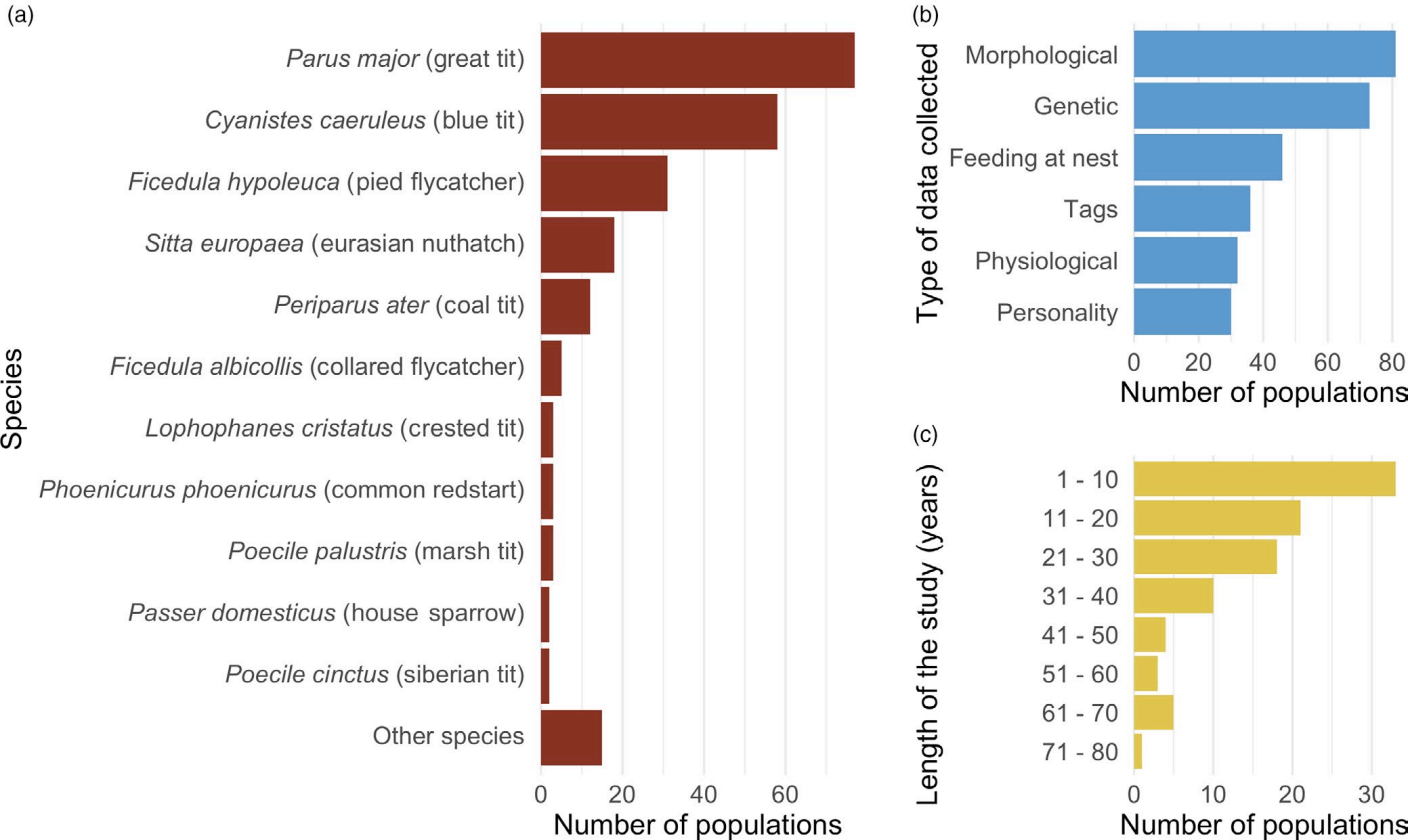
Summary information on the number of populations hosted at SPI‐Birds that (a) collect data on a certain species; (b) collect different types of data on individuals (alongside basic breeding parameters); (c) have been studied for a certain period of time

To date, we count more than 120 members from 21 countries, monitoring over 80 populations of 19 species (Figures 1 and 2a). Currently, the majority of the species are hole‐nesting passerines (Table 1), but as a part of our long‐term goal, we are actively reaching out to researchers who work on other species groups across the world, and so far have incorporated some of these into the database (e.g. owls, seabirds, dunnocks). The only requirement that needs to be met is that most of the birds in a population can be individually recognized (which is commonly achieved by a metal or coloured rings) and that at least one component of the breeding success of these individuals (e.g. laydate, clutch size) has been measured over at least 2 years.

**Table 1:** Number of unique breeders, breeding attempts and ringed nestlings per species, as hosted at SPI‐Birds database

Species	No. unique breeders	No. breeding attempts	No. ringed nestlings
*Parus major*	60,501	90,882	590,157
*Cyanistes caeruleus*	44,878	59,386	438,840
*Ficedula albicollis*	35,088	45,933	247,141
*Ficedula hypoleuca*	22,406	26,099	116,662
*Sterna hirundo*	3,063	10,097	NA
*Poecile montanus*	3,000	3,673	12,945
*Poecile palustris*	2,319	1,537	3,495
*Periparus ater*	1,462	2,160	13,662
*Passer domesticus*	1,021	2,690	2,890
*Corvus monedula*	852	2,120	NA
*Prunella modularis*	305	466	432
*Lophophanes cristatus*	195	164	465
*Emberiza melanocephala*	186	66	221
*Poecile cinctus*	184	253	NA
*Sitta europea*	133	743	456
*Parus minor*	120	232	745
*Strix aluco*	63	84	168
*Phoenicurus phoenicurus*	45	487	1,586
*Parus varius*	10	24	76
Overall	175,831	247,096	1,416,293

### 1.4 Community data standards

To facilitate data compatibility and integration, SPI‐Birds has already created data standard for storing breeding‐season data on individually monitored birds. This standard format is described in detail on the SPI‐Birds GitHub repository (Culina et al., 2019). It is designed to cover the data fields that are common across most individual‐based bird studies, and is aligned with the standards suggested by the Ecological Meta‐Data Language (EML, Jones et al., 2019) and the principles of FAIR data (Wilkinson, 2016). The standard format is dynamic and can be further extended or adjusted to accommodate the breeding biology (e.g. cooperative breeders) of species yet to be included into the database.

SPI‐Birds creates tailored pipelines to convert data from each research group/contributor (i.e. primary data format, Box 2) into the standard format. We hope that this standard format will be adopted by both new and existing research groups to archive their data. The existing groups will be more likely to start using the standard format once their old data have been converted into it by SPI‐Birds. We further plan to extend this format (and create new standards) to accommodate other information (e.g. genetic, hormonal, colouration, ecotoxicological, behavioural data). Currently, each population's meta‐data clearly indicate whether this additional information has been collected, and the corresponding data can be stored at SPI‐Birds (although not yet standardized). For example, physiological or personality data has been collected in almost 30 populations (Figure 2b).

### 1.5 Data processing: Integration, quality checks, yearly updates

**Figure 3:**
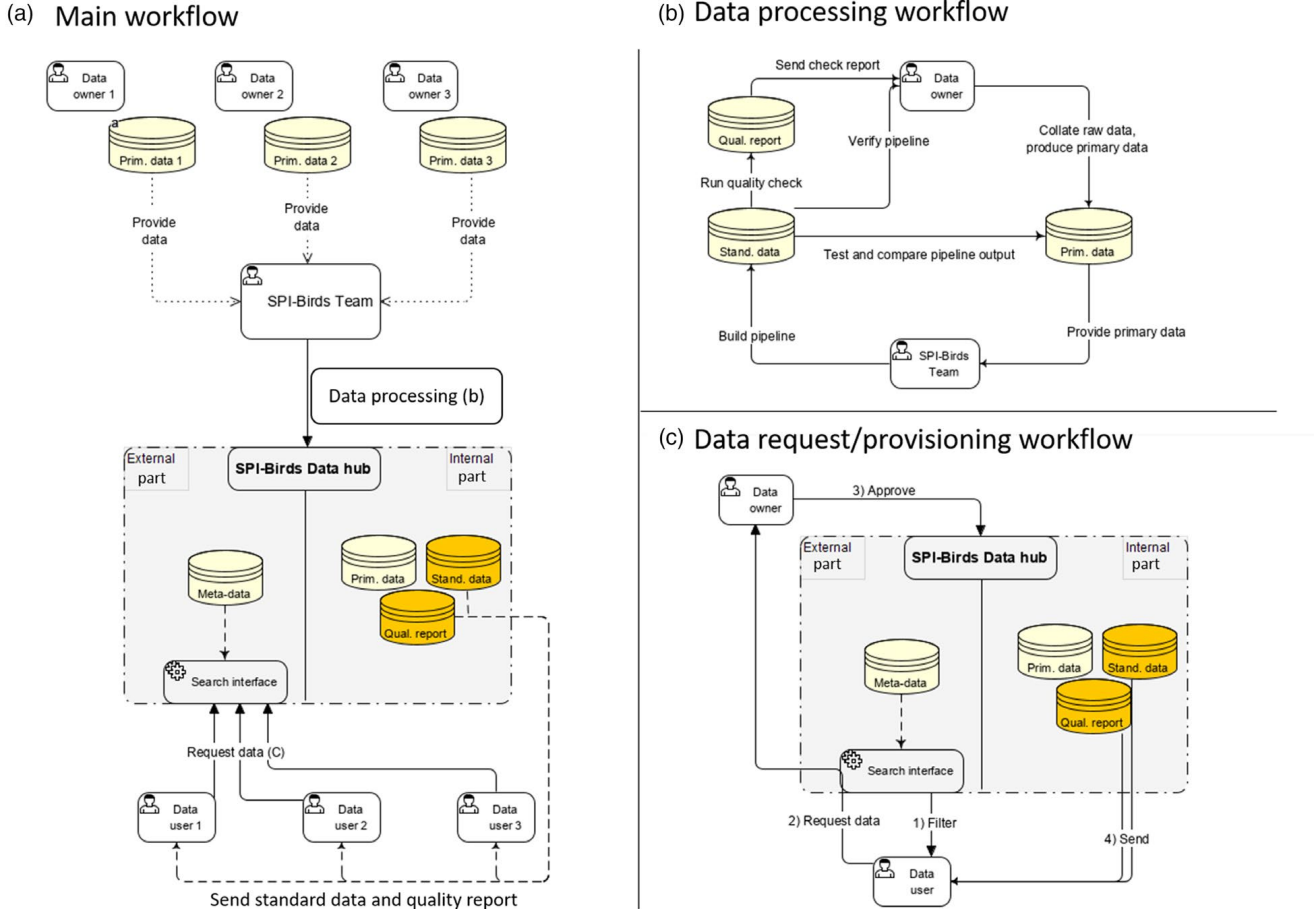
Overview of SPI‐Birds infrastructure. (a) Main data workflow that consists of provisioning of primary data, data processing (standardization and quality checks) and data request and provisioning. Panel (b) describes data processing, and panel (c) describes data request and provisioning process. The internal part (not accessible for users) of the SPI‐Birds data hub stores versioned data in the primary and the standard format, with an accompanying quality report for each dataset. Users can search meta‐data and request data (c) via the external part of the SPI‐Bird data hub (i.e. website). Data are sent to the user (if approved by the data owner, or if data are fully open access) in the community standard format, together with the data‐quality report(s). Prim. data = data in the primary format, as stored by a data owner; Stand. data = data in the standard format; Qual. report = a report produced by the standard quality check

Figure 3a provides an overview of the SPI‐Birds data flow (data collection, standardization, request and provisioning). First, data owners upload their primary data (Box 2) to SPI‐Birds. Primary data come in various storing formats of different complexity, from spreadsheet files (e.g. MS Excel) and simple self‐contained databases (e.g. MS Access), to dedicated database servers (e.g. MySQL). Tailored pipelines are then constructed (Figure 3b) for each dataset to convert primary data into the standard format. The pipeline code is discussed with the data owners (e.g. discussion of how fields in the primary data are coerced into corresponding fields in the standard format) to ensure maximum accuracy. Pipeline construction sometimes requires several iterations before an accurate pipeline is created. At this point, the pipeline can be confidently applied. The primary data and data in the standard format are stored within the secure SPI‐Birds data hub (Figure 3a), at the file server cluster of the Netherlands Institute of Ecology (NIOO‐KNAW) and backed up seven times a week. The pipelines are publicly available via GitHub.

S*tandard quality check* is applied to the standardized data. It involves automated checks for missing data, formats of variables (e.g. date, integer), inconsistencies between variables (e.g. false brood assignment) and unexpected values within variables. The output of the standard quality check are two types of flags: ‘warnings’ (i.e. values that are uncommon or unusual) and ‘likely errors’ (i.e. values that should be impossible). In discussion with the data owner, warnings and likely errors are resolved, if at all possible, and the quality check is updated. If data owners decide to address the ‘warnings’ and ‘likely errors’ and update their own primary data, these updated primary data will then be stored in the SPI‐Birds data hub, under the version control system. Finally, any remaining unresolved flagged records appear in the quality check report that is sent to the user. The ‘warnings’ and ‘likely errors’ list is part of the meta‐data for each version of the dataset.

For all populations with ongoing data collection primary data are updated to a new version every year and may include not only additional data collected over the additional year but also corrections of errors found in earlier data. We store all versions of the primary data following these yearly updates. This way, we aid to the reproducibility of results based on the version of the data used for the analysis.

### 1.6 Data use: Discovery, provisioning, terms of use

Populations hosted at SPI‐Birds can be searched via SPI‐Birds website based on meta‐data (e.g. species studied, country, length of data collection, variables measured). Once the relevant populations have been identified, data can be requested using the SPI‐Birds request form (Figure 3c). Unless the data owners have made their data fully open access, data requests are sent for approval to the data owner. If approval is given, standardized data from the requested population(s), accompanied by the standard quality check report, are delivered to the user, and the data owner is informed about the data sharing. When the user requests multiple datasets, all datasets (in a standard format) are compiled and sent to the user. Each dataset comes with a specific terms of data use, and these are stated in its meta‐data. To give credit to those who have been collecting/managing the raw data, as a minimal requirement for data use (i.e. even when the data owner does not request any other conditions of data use) we ask that the data owner(s) and funding source(s) that they state in the meta‐data, are explicitly acknowledged upon data use (e.g. in the acknowledgment section of an article). We also require acknowledgment of the SPI‐Birds Network and Database, and citation of this paper. Furthermore, SPI‐Birds encourage citation of the dataset source (or related publication) via DOIs (digital object identifiers). The detailed Data Access Policy can be found on our website.

### 1.7 Understanding data and their limitations

As discussed above, datasets come with errors and limitations. While SPI‐birds increases data integrity and quality, the standard data may still contain errors, and data from different populations might still not be entirely comparable. To enable users to understand how the primary data and standardized data were derived and to highlight potential limitations in the dataset, we provide several documents (as a part of the meta‐data). These include the description of the study site (e.g. location, size, habitat type), data collection protocols and the list of any initial quality checks on the primary data conducted by a data owner. This way, users can better understand how the primary data were derived. Next, we provide a detailed description of decisions and assumptions made during the conversion of data from primary to standard format (with all the pipelines openly available via GitHub), details on quality checks conducted by SPI‐Birds and the resulting quality report. Finally, we publish a series of ‘technical reports’ on the SPI‐Birds website, where we discuss a range of topics related to methodological conventions (e.g. conversion from one type of tarsus measurement method to another type) and potential biases induced by methodological approaches to data collection (e.g. impact of the frequency at which nests are checked on the estimation of laying date, the impact of nest box design on the vital rates, such as survival of young, Lambrechts et al., 2010).

### 1.8 Lessons learned—Creating a community data hub

The need to adopt global meta‐data and data standards in ecology and evolution is growing (Poisot et al., 2019; Schneider et al., 2019). We strongly believe, and our example supports, that the best way to achieve the adoption of global standards is to first create standards for well‐defined communities (Poisot et al., 2019). When research communities that work on a similar type of data have established their own standards, it becomes easier to scale up to even larger, more global standards (e.g. EML, Jones et al., 2019). Lessons learned from the SPI‐Birds example can be useful to research communities where many researchers (groups) collect data of a comparable type (or purpose), but where research protocols and data management are not uniform (i.e. the long‐tail of science, Box 2, Palmer et al., 2007; Wallis et al., 2013).

We suggest four key points in establishing a common database and community data standards in the long‐tail of science: (a) *How to start*: Aim to identify researchers/groups that belong to your research community. This is largely a snowballing process—once you locate several members, ask them to identify others. Ideally, organize a kick‐off meeting to discuss the aims, distribution of tasks and further steps. From our experience, it is important to have at least several research groups keen on the project at the start. Furthermore, it is important to consider needs and fears of your research community when deciding on the best working model. For example, our success in mobilizing members largely comes from a decentralized approach; data owners keep full control (i.e. ownership) over their data, and over their data management practices. (b) *Keep the community engaged and informed*: We found it essential to enable all the members to have the opportunity to contribute to the decisions made. For example, all of our members can provide feedback on any component of the project. Second, it is important, especially at the start, to show that the project is active. We suggest publishing a newsletter every month or two, and creating a social media account. We tweet about each data set we receive, keeping the community informed of our continuous growth. Third, organize workshops/meetings where the community physically (or virtually) comes together. (c) *Funding*: Plan to allow for different funding scenarios. We find that it is best to plan finances in steps (if no long‐term large funding is available at the very start). Make sure that the first step—what you want to achieve at the minimum—is financially covered at the start. This must include securing a permanent, long‐term platform to archive the datasets. After that, plan in five‐year (or similar) steps. Here make sure that in the worst‐case scenario (no further funding secured) each step is maintainable with a minimal financial and personnel commitment. For example, our first step was to integrate data on hole‐nesting passerines in Eurasia, and this period was financially covered by a grants held by participating individuals, and volunteer contributions from several members. After this initial phase, the SPI‐Birds database can be kept functional with a minimal investment (e.g. storage capacity). In the next step, we plan to increase our scope. At this stage, our project has already proven successful, which makes it more attractive for longer term support (e.g. European open science funds). Finally, we ask (but do not mandate) that those whose research plans rely on the collective power of datasets hosted at SPI‐Birds to allocate some of their resources to the SPI‐Birds initiative.

### 1.9 Vision for an integrated future

SPI‐Birds is a large‐scale initiative that integrates data on individual‐based studies of breeding birds and connects researchers who collect data in these populations. With this paper we also call for additional members to join our fast growing community. To join, please use the contact details as given on the SPI‐Birds website (www.spibirds.org). We are inviting contributions from anyone who monitors a population of a bird species, where birds are individually recognizable (usually this would be numbered or colour rings), and where breeding success (at least one component of the breeding success, e.g. clutch size) is recorded over years (at least 2 years). In further developments of the database we plan to: (a) cover additional populations, species and a wider geographical area; (b) integrate and standardize other data types (e.g. hormonal, behavioural); (c) connect with ongoing centralized efforts to map the full spectrum of different types of data on birds that can complement each other. Here the main collaborators are scientific groups that centralize the collection of complementary types of data (e.g. Movebank, Fiedler & Davidson, 2012; Kranstauber et al., 2011, EURING, du Feu et al., 2016; the great tit HapMap project, Spurgin et al., 2019). Within this scope, we can connect individual‐level data hosted at SPI‐Birds to other types of data on the same individuals based on their unique ID and provide even more comprehensive information on individuals across their full life cycle. A second target group are citizen science projects such as Nestkast (de Jong et al., 2018), or the Woodland Trust phenology network (https://naturescalendar.woodlandtrust.org.uk/).

SPI‐Birds can also serve as a platform to enable better resource allocation between research groups. For example, while a data owner might have the data, they might lack funds to analyse them. On the other hand, a data user might have funds or even apply for funds based on these data. SPI‐Birds can thus help pull the resources (data and funds) together, thereby enabling scientific projects, and progress, where it may otherwise be unlikely to occur. We also encourage use of SPI‐Birds data in student projects. Finally, during the unforeseen international crisis, such as caused by a novel corona virus during writing of this contribution, SPI‐Birds provided an excellent platform to update and document field situations and to mitigate the unbalanced effects of the crisis on research groups. We hope that initiatives such as SPI‐Birds can truly help a transition to a new level of ecological synthesis.

## AUTHORS' CONTRIBUTIONS

A. Culina, F.A., L.D.B., M.D.B., A. Charmantier, E.F.C., T.E., E.M., C.R.N., B.C.S., B.‐E.S., S.J.G.V., M.E.V. conceptualized the ideas; A. Culina wrote the manuscript draft and supervised the project; Z.Z., A. Culina and S.M.D. produced the Figures; A. Culina and M.E.V. enabled overall data curation. All the co‐authors collected datasets hosted at SPI‐Birds, critically reviewed and edited the manuscript.

## Data Availability

Data and code to recreate Figure 2 are available from Dryad Digital Repository https://doi.org/10.5061/dryad.51c59zw6r (Culina et al., 2020).
